# Lipid Droplets in Disease

**DOI:** 10.3390/cells8090974

**Published:** 2019-08-26

**Authors:** Paul Dalhaimer

**Affiliations:** Department of Chemical and Biomolecular Engineering, University of Tennessee, Knoxville, TN 37996-2200, USA; pdalhaim@utk.edu

Lipid droplets (LDs) are a crucial part of lipid storage; thus, they are important players in a variety of diseases that are affected by lipid imbalances such as obesity, fatty liver disease, type 2 diabetes, Alzheimer’s disease, cardiovascular disease, and cancer. In addition, they play important roles in pathogen-host interactions. In this issue of *Cells*, both original research and reviews highlight the new advances being made in the role of lipid droplets in disease. 

LDs vary in composition by cell type but in mammals they are comprised mostly of the neutral lipids triacylglycerol (TAG) and esterified cholesterol (CE). With the exception of foam cells, which have CE LDs [1], the main cells of interest in lipid-imbalance-diseases such as adipocytes, hepatocytes, and myocytes have mostly TAG LDs. TAG is synthesized in the endoplasmic reticulum (ER) and in/on the droplets themselves from the neutral lipid precursor diacylglycerol (DAG) [2]. It is hypothesized that LDs form from the outer leaflet of the ER when the local concentration of neutral lipids is high enough to form a lens of TAG (and possibly also DAG) [3]. Although TAG is often the most discussed neutral lipid comprising LDs, DAG has been shown to play a crucial role in physiology [4]. In this issue, Pruess and coworkers developed a liquid chromatography triple quadrupole mass spectrometry (LC-MS/MS)-method for the rapid quantification of DAG species in tissue samples [5]. Interestingly, the authors found elevated levels of DAG and ceramide in hepatocyte, heart, and muscle LDs in a mouse model of non-alcoholic fatty liver disease (NAFLD). Both DAG and ceramide will continue to be intriguing players in their role in mammalian health in the context of LDs and lipid metabolism. Another interesting molecule in lipid metabolism is retinoic acid. Bobowski-Gerard and coworkers describe the role of retinoic acids in liver injury [6]. They propose new approaches that will better elucidate how retinoic acid is generated by the liver during LD loss in hepatic stellate cells.

After formation, LDs can increase in size by fusion or by inhibited TAG breakdown mechanisms. LD fusion plays a large role in LD function. Cell death-inducing DNA fragmentation factor alpha (DFFA)-like Effector (CIDE) proteins are important players in LD fusion [7]. Slayton and coworkers review the physiological roles and metabolic pathways regulated by CIDE proteins [8]. CIDE is one of many factors bound to LDs that affect the role of LDs in physiology [9]. In addition to fusion, there has been much focus on LD-bound factors that are responsible for TAG lipolysis. These include perilipins (PLINs), alpha-beta hydrolase domain 5 (ABHD5/CGI-58), hormone sensitive lipase (HSL), monoacylglycerol lipase (MGL), and adipose triglyceride lipase (ATGL). These factors work in concert to regulate TAG lipolysis [10,11,12]. Missaglia and coworkers review how disruptions in ATGL and ABHD5/CGI-58 cause neutral lipid storage diseases [13]. Perilipins play a crucial role in LD size by protecting TAG in the LDs from ATGL action. Listenberger and colleagues show that the composition of the LD phospholipid monolayer plays a key role in this process [14]. They found a decrease in the ratio of surface phosphatidylcholine (PC) to phosphatidylethanolamine (PE) in the hepatic LDs in rats with alcoholic liver injury. Perilipin 2 (PLIN2) levels also increased on the surfaces of the LDs, which should hamper TAG processing. Furthermore, reducing PC:PE ratios on liposomes increased the binding of PLIN2 to their surfaces in vitro. By also decreasing the LD PC:PE ratio in cell culture, the authors detected increased association of PLIN2 to LDs. Their work shows important links between LD surface phospholipids and PLIN2 recruitment to LDs.

It is widely appreciated that LDs interact with other organelles including mitochondria [15]. Of relevance here is the beta-oxidation of fatty acids. Chokchaiwong and coworkers explore the genotype-phenotype relationship of electron-transfer flavoprotein dehydrogenase gene (*ETFDH*) with the pathogenesis of multiple acyl-CoA dehydrogenase deficiency (MADD) in mutated lympholastoid cells [16]. They found that MADD patients have increased LDs. Riboflavin and/or coenzyme Q10 supplementation rescues cells from this LD accumulation. Their results help clarify the molecular pathogenesis of MADD.

Understanding the role of LDs in disease provides pathways for treatment of obesity, NAFLD, and type 2 diabetes. Chen and coworkers explore the mechanisms of urodeoxycholic acid treatment, which has been shown to possess antioxidant and anti-inflammatory properties and also alleviates mitochondrial dysfunction and the progression of obesity-related diseases [17]. In this issue, they show that urodeoxycholic acid decreases LD number and size, reduced free fatty acid (FFA) and TAG levels, improved mitochondrial function, and enhanced white adipose tissue (WAT) browning in *ob/ob* mice. Importantly, they found that urodeoxycholic acid acts to reduce whole body adiposity.

LD presence in skeletal muscle is a hallmark of insulin resistance in type 2 diabetes mellitus. Paradoxically, trained athletes accumulate lipids in skeletal muscle and the size of their LDs in muscle tissue is positively correlated with insulin sensitivity. Li and coworkers review this phenomenon, which is called the athlete’s paradox [18].

To understand how LD-bound lipase expression affects TAG homeostasis in human hepatocellular carcinoma (HCC) cells, Berndt and colleagues built a model, which shows that minor cell-to-cell variation in the expression level of these lipases can give rise to significant variations in LD size distributions [19]. In particular, they found that HCCs can be categorized into two groups based on their rate of free fatty acid uptake, phospholipid synthesis, and very low-density lipoprotein (VLDL) synthesis. Also, TAG accumulation in HCC did not correlate with the uptake rate of free fatty acids. These results point to important metabolic subpopulations of hepatocytes in HCC. 

It is now being appreciated that lipid imbalance plays a major role in neurodegeneration such as in Alzheimer’s disease. The E4 allele of *APOE*, whose gene product is a major structural component of low-density lipoproteins (LDLs), is a strong genetic risk factor for the development of late onset Alzheimer’s disease [20]. Farmer and coworkers show that E4 expression in astrocytes results in an increase in small LDs compared to astrocytes expressing E3 [21]. PLIN2 levels were increased in the astrocytes expressing E4 over the E3-expressing cells. They also found that E4 astrocytes had decreased uptake of palmitate and decreased oxidation of exogenously supplied oleate and palmitate. Their work points to important links between the *APOE* gene and lipid metabolism in neurodegeneration. Lastly, Libbing and coworkers review the literature on the importance of LDs in host-pathogen interactions with a focus on bacteria [22]. The contributions to this issue show that LD formation, stability, and breakdown continue to be recognized as important players in a variety of diseases. In the next decade we will begin to see progress in the treatments of these diseases through LD-targeting strategies. 
